# Supplementary material for: Chronologically sampled flight feathers permits recognition of individual molt-migrants due to varying protein sources
Source: PeerJ. 2015 Jan 22;3:e743. doi: 10.7717/peerj.743 (PMC4312066; doi:10.7717/peerj.743)
Supplement: Supplemental Information 1 — The left section of this file presents raw data for nitrogen and carbon at 5 mm sampling distances. The right section presents the raw data for nitrogen at fine sampling intervals, of 1 or 2 mm for the same three geese. [file peerj-03-743-s001.pdf]

Course sampling

Chronology	Goose 501	Goose 501	Goose 508	Goose 508	Goose 509
mm from tip	N isotope Ratio	C isotope ratio	N isotope Ratio	C isotope ratio	N isotope Ratio
5	7.61	-26.34	8.87	-26.59	7.39
10	7.78	-26.29	8.81	-26.56	7.47
15	7.88	-26.23	8.14	-26.49	7.32
20	7.88	-26.12	8.21	-26.57	7.7
25	7.8	-26.49	7.96	-26.47	7.58
30	7.76	-26.42	7.98	-26.55	7.39
35	7.73	-26.56	7.88	-26.82	7.4
40	7.66	-26.44	7.96	-26.63	7.33
45	7.55	-26.32	7.75	-26.76	7.38
50	7.85	-26.12	7.9	-26.32	7.76
55	7.88	-26.01	7.86	-26.9	7.85
60	7.53	-25.45	7.69	-26.75	7.69
65	7.38	-25.88	7.84	-26.45	7.71
70	7.5	-26	7.81	-26.73	7.57
75	7.51	-25.86	7.66	-26.53	7.67
80	7.85	-25.84	7.67	-26.87	7.66
85	7.63	-25.96	7.55	-26.66	8.66
90	7.78	-26.14	7.62	-27.03	7.92
95	7.69	-26.42	7.48	-26.41	7.67
100	7.72	-26.25	7.37	-26.15	7.7
105	7.51	-26.16	7.39	-26.9	7.56
110	7.56	-25.88	7.26	-26.99	7.38
115	7.36	-25.51	7.22	-27.01	7.44
120	7.33	-25.59	7.23	-26.74	7.27
125	7.36	-24.88	7.29	-26.17	
130	7.64	-25.88			
135	7.37	-25.74			
140	7.25	-25.5			
145	7.07	-26.05			

Goose 509 C isotope ratio	Fine Sampling Chronology mm from tip	Goose 501 N isotope Ratio	Chronology mm from tip	Goose 508 N isotope Ratio
-26.54	1	8.18	6	8.18
-26.16	2	8.06	8	8.4
-26.16	3	7.94	10	8.48
-26.22	4	7.99	12	8.24
-26.15	5	7.72	14	8.29
-26.37	6	7.8	16	8.19
-26.22	7	7.91	18	8.17
-25.97	8	7.81	20	8.26
-26.15	9	7.75	22	8.35
-26.02	10	7.65	24	8.14
-26.52	11	7.6	26	8.14
-26.34	12	7.7	28	8.12
-26.37	13	7.51	30	8.09
-26.31	14	7.65	32	8
-26.01	15	7.58	34	8.05
-25.63	16	7.2	36	8.19
-24.75	17	7.4	38	8.2
-25.96	18	7.25	40	8.19
-25.93	19	7.47	42	7.94
-25.85	20	7.2	44	8.14
-26.03	21	7.41	46	8.11
-26.24	22	7	48	8.1
-26.25	23	7.21	50	8.15
-26	24	6.96	52	7.81
	25	7.04	54	7.79
	26	6.93	56	7.97
	27	6.84	58	8.03
	28	6.81	60	7.95
	29	7.03	62	7.9
	30	6.85	64	7.93
	31	6.91	66	7.93
	32	6.8	68	7.6
	33	6.72	70	7.64
	34	6.82	72	7.99
	35	6.73	74	7.8
	36	6.74	76	7.63
	37	6.54	78	7.84
	38	6.55	80	7.93
	39	6.64	82	7.89
	40	6.68	84	7.8

41	6.66	86	7.81
		88	7.38
		90	7.66
		92	7.5
		94	7.63
		96	7.71
		98	7.41
		100	7.52
		102	7.51

Chronology	Goose 509
mm from tip	N
3	7.62
4	7.68
5	7.83
6	7.62
7	7.72
8	7.81
9	7.61
10	7.55
11	7.5
12	7.71
13	7.92
14	7.71
15	7.48
16	7.49
17	7.63
18	7.55
19	7.33
20	7.64
21	7.4
22	7.64
23	7.79
24	7.74
25	7.33
26	7.89
27	7.63
28	7.55
29	7.57
30	7.57
31	7.67
32	7.52
33	7.72
34	7.69
35	7.67
36	7.53
37	7.61
38	7.69
39	7.13
40	7.49
41	7.53
42	7.54

43	7.62
44	7.61
